# Persistence of an extracellular systemic infection across metamorphosis in a holometabolous insect

**DOI:** 10.1098/rsbl.2017.0771

**Published:** 2018-02-07

**Authors:** David F. Duneau, Brian P. Lazzaro

**Affiliations:** 1 Department of Entomology, Cornell University, 129 Garden Avenue, Ithaca, NY 14853, USA; 2 Université Toulouse 3 Paul Sabatier, CNRS, ENSFEA; UMR5174 EDB (Laboratoire Évolution & Diversité Biologique), 31068 Toulouse, France; 3 Cornell Institute of Host Microbe Interactions and Disease, Cornell University, Ithaca, NY, USA

**Keywords:** bacterial infection, transmission across host life stages, extracellular bacteria, *Drosophila*, *Providencia rettgeri*, host–parasite interaction, transstadial transmission

## Abstract

Organisms with complex life cycles can differ markedly in their biology across developmental life stages. Consequently, distinct life stages can represent drastically different environments for parasites. This difference is especially striking with holometabolous insects, which have dramatically different larval and adult life stages, bridged by a complete metamorphosis. There is no *a priori* guarantee that a parasite infecting the larval stage would be able to persist into the adult stage. In fact, to our knowledge, transstadial transmission of extracellular pathogens has never been documented in a host that undergoes complete metamorphosis. We tested the hypothesis that a bacterial parasite originally sampled from an adult host could infect a larva, then survive through metamorphosis and persist into the adult stage. As a model, we infected the host *Drosophila melanogaster* with a horizontally transmitted, extracellular bacterial pathogen, *Providencia rettgeri*. We found that this natural pathogen survived systemic infection of larvae (L3) and successfully persisted into the adult host. We then discuss how it may be adaptive for bacteria to transverse life stages and even minimize virulence at the larval stage in order to benefit from adult dispersal.

## 1. Introduction

Organisms with complex life cycles can differ dramatically in their biology across developmental life stages. Consequently, distinct life stages can represent drastically different environments for parasites. The differences between developmental stages are especially striking in holometabolous insects. The larvae of holometabolous insects are dedicated to growth and development, whereas adults are generally more focused on reproduction. Larval versus adult insects often additionally have different feeding behaviours and nutrient sources. These distinctions in physiological demands and resource availabilities can contribute to differences in immune capability [1] and suitability for parasite development. Parasites able to infect and survive in one life stage may not be successful in another life stage. The transition between life stages (i.e. metamorphosis) may be particularly challenging for the parasite and might serve as a block on parasite development and/or transmission. This could be particularly relevant for extracellular parasites that are not transmitted vertically, yet to our knowledge, there has been no study investigating the possibility that a systemic infection by an extracellular, horizontally-transmitted pathogen can persist through metamorphosis, a phenomenon called transstadial transmission.

Transstadial transmission of bacteria is a key process in some vector-borne diseases. For example, *Borrelia burgdorferi*, the bacterium causal for Lyme disease, is ingested with a blood meal by its vector ticks in an early developmental stage, migrates into the salivary glands and is transmitted to subsequent hosts in a later developmental stage [2]. Hence, it is crucial for the bacterium to be able to survive the moulting events of the vector host. However, if bacterial transstadial transmission is well known in arachnid vectors (e.g. ticks and mites, hosts that carry little differences between stages), bacterial transstadial transmission is less known in holometabolous insects, where the differences are drastic. Transstadial persistence of vertically transmitted intracellular (e.g. *Wolbachia* and microsporidian species) and extracellular (e.g. *Spiroplasma* species) bacteria has been described in holometabolous insects that undergo complete metamorphosis [3–5], but it is as yet untested whether the same phenomenon can occur in the context of horizontally acquired opportunistic infections.

We infected larvae of the holometabolous insect *Drosophila melanogaster* with its natural bacterial pathogen *Providencia rettgeri* [6] and asked whether this exclusively horizontally transmitted extracellular bacteria could survive metamorphosis and persist into the adult stage. Our strain of *P. rettgeri* was isolated from an adult host captured in the field and we have worked with it extensively in the laboratory [7–9], so we know it is capable of infection and growth in the adult, up to a very large density at the death of the host [10]. *Providencia rettgeri* is a ubiquitous, opportunistic pathogen. It has been isolated from diverse abiotic environments, such as fresh water sources, run-off wastewater and explosive-contaminated soil [11,12], and biotic environments including holometabolous insects (e.g. true fruit fly (*Dacus* species), stable fly (*Stomoxys calcitrans*), *Drosophila*, Mexican fruit fly (*Anastrepha ludens*)), where it lives as either a commensal or a pathogen [6,13–15]. Interestingly, there is some evidence that *P. rettgeri* produces organic compounds that attracts screwworm flies (*Cochliomyia hominivorax*), which suggests that it could use this Dipteran as vector for dispersal [16]. *Drosophila melanogaster* has well described stages and a well-characterized immune response [17]. There is a spike in expression of antimicrobial defence genes during the transition between larval and pupal stages, possibly linked to hormonal changes inducing pupation [18,19]. This spike in defence gene expression could reduce bacterial survival. Yet we find that *P. rettgeri* infecting *D. melanogaster* larvae survive metamorphosis and successfully persist into the adult host. We believe this is the first report of transstadial transmission of systemic extracellular bacterial infection in a holometabolous insect, and we speculate that the occurrence of this phenomenon is underestimated in the wild and could potentially permit dispersal of bacteria among ephemeral microenvironments, such as rotting fruits.

## 2. Material and methods

### (a) Fly husbandry and infection

Canton S larvae were raised on glucose-yeast medium (82 g l^−1^ yeast, 82 g l^−1^ glucose, 1% *Drosophila* Agar) at approximately 25°C with a 12 h/12 h dark/light cycle. Injections of 23 nl of bacterial suspension were performed at the L3 stage. The suspension was either composed of the bacterial culture media (LB broth) or of approximately 3000 bacteria suspended in culture media (A_600_ = 0.1 of *P. rettgeri* in Miller LB [20]). We also had control larvae that were not injected. Adult survival was checked daily over 90 days. Upon death, adults were checked for persistence of infection by homogenizing individual flies in PBS, spreading the homogenate on an LB/agar plate and scoring colony formation after overnight incubation at 37°C.

### (b) Statistical analyses

Statistical analyses were performed with R [21]. Differences in the proportion of successful host pupal emergence were first tested with a generalized linear model (glm) with a binomial distribution of the error. Then, we tested pairwise differences with Pearson's Chi-squared tests. The difference in time to emergence was tested with a Cox proportional hazard model (*coxph* function in the Survival package [22]) and plotted as 1-proportion of occurred event. To compare survival between treatments, we used a Cox proportional hazard model (*coxph* function in the Survival package [22]).

## 3. Results

**Figure 1.**
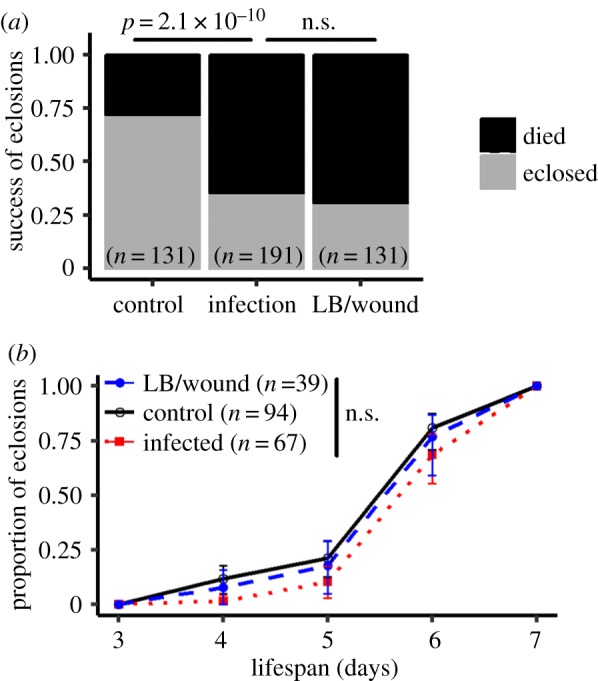
Effect of larval systemic infection on larval life-history traits. (*a*) Success of eclosion of larvae infected with *P. rettgeri* in comparison with controls. The procedure of injection affected the probability of survival but the presence of live bacteria had no additional effect. (*b*) Timing of eclosion of larvae infected with *P. rettgeri* in comparison with controls. Among the larvae that eclosed, the infection did not increase the time of eclosion.

The rate of adult emergence was significantly lower for larvae that were injected with *P. rettgeri* suspension or injected with sterile LB than control larvae on which no injection was performed (glm with binomial distribution: 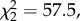 *p* < 0.0001, figure 1*a*). However, the injection of live bacteria did not lower the probability of adult emergence compared to injection of sterile medium (Pearson's chi-squared test: 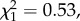 *p* = 0.46). We did not detect an effect of injection on the time to emergence (Coxph: 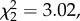 *p* = 0.22, figure 1*b*), although there was a non-significant tendency for infected larvae to develop more slowly than control larvae.

**Figure 2.**
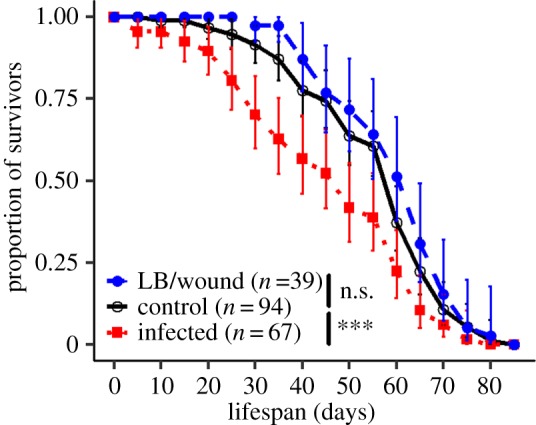
Effect of larval systemic infection on adult lifespan. Infection during the larval stage significantly reduces adult lifespan.

Adults that had been infected with *P. rettgeri* as larvae retained their infections through metamorphosis and into adulthood 100% of the time. Injection of sterile bacterial growth medium did not reduce adult lifespan relative to uninjected controls (*z* = −0.8, exp(coef)= 0.85, *p* = 0.41). However, the persistent infection reduced adult lifespan relative to controls (Coxph: 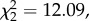 *p* = 0.002, figure 2). Only 42% of infected individuals survived more than 50 days compared to 64% of uninjected controls and 71% of sham-injection controls (infected versus uninjected control: *z* = 2.97, exp(coef)= 1.61, *p* = 0.003). Data can be found in electronic supplementary material, file S1.

## 4. Discussion

Our results show that extracellular bacteria, such as *P. rettgeri,* can infect the larval stage of *D. melanogaster* and persist through metamorphosis to remain viable in the adult host. The infection at the larval stage with our experimental bacterium, *P. rettgeri*, did not impose a detectable cost to the host at larval stage. It neither caused a decreased probability of successful metamorphosis (figure 1*a*) nor a delayed developmental time (figure 1*b*). Moderate cost on lifespan was detected on persistently infected adults, though, with a modest decrease in maximum lifespan and a sharp decrease in median time to death (figure 2).

This work highlights the possible general role of the host life cycle in the evolution of host–parasite interactions. The persistence of infection throughout life stages can have implications for the evolution of epidemiology and bacterial virulence. Flying adults of holometabolous insects obviously differ in their dispersal capability relative to crawling larvae, and the ability to remain inside the host through metamorphosis might increase parasite dispersal. Larvae and adults also differ in their physiology, immunological status and nutritional resource availability, which could pose a challenge for transstadial transmission of parasites. Documented examples of horizontally transmitted parasites surviving across life stages of holometabolous insects are rare. However, some parasites with complex life cycles need to transit multiple hosts or multiple host life stages in order to ensure transmission (e.g. Gordian worms that need to go from water to the ground go through the life cycle of their aquatic host, such as damselflies, which leave water when adults [23]). The existence of such parasites supports the idea that natural selection may sometimes favour pathogens able to survive metamorphosis. Our results demonstrate that such survival can indeed occur.

Furthermore, in order to reach and survive metamorphosis, the pathogen must not kill the host during development. Thus, we expect that selection would favour parasites of larvae that exhibit little virulence until they reach the adult host stage. On the contrary, parasites that are certain to be eliminated during metamorphosis might be predicted to be virulent at larval stages. Further studies are needed to make the link between parasite dispersal, capacity to infect all host stages and evolution of stage-specific virulence. Nevertheless, the possibility that an opportunistic extracellular pathogen can persist through life stages of *Drosophila*, coupled with the low cost of larval infection, suggests that such a scenario is plausible. Considering that holometabolism is a characteristic of at least 45% of all known animal species [24], natural selection for transstadial transmission could have major consequences for shaping the evolution of opportunistic pathogens as a whole.

## Supplementary Material

Data file

## Data accessibility

See electronic supplementary material, file S1.
